# P2X3 purinergic receptor overexpression is associated with poor recurrence-free survival in hepatocellular carcinoma patients

**DOI:** 10.18632/oncotarget.6240

**Published:** 2015-10-26

**Authors:** Janielle P. Maynard, Ju-Seog Lee, Bo Hwa Sohn, Xiaoying Yu, Dolores Lopez-Terrada, Milton J. Finegold, John A. Goss, Sundararajah Thevananther

**Affiliations:** ^1^ Department of Pediatrics, Division of Gastroenterology, Hepatology and Nutrition, Texas Children's Liver Center, Houston, TX, USA; ^2^ Program in Translational Biology and Molecular Medicine, Baylor College of Medicine, Houston, TX, USA; ^3^ Department of Systems Biology, UT MD Anderson Cancer Center, Houston, TX, USA; ^4^ Department of Medicine, Division of Gastroenterology, Baylor College of Medicine, Houston, TX, USA; ^5^ Department of Pathology and Immunology, Baylor College of Medicine, Houston, TX, USA; ^6^ Department of Surgery, Baylor College of Medicine, Houston, TX, USA

**Keywords:** hepatocellular carcinoma, cell-cycle, extracellular ATP, purinergic receptors, JNK signaling

## Abstract

P2 purinergic receptors are overexpressed in certain cancer tissues, but the pathophysiologic relevance of purinergic signaling in hepatocellular carcinoma (HCC) remains unknown. To examine the role of P2 purinergic signaling in the pathogenesis of HCC and characterize extracellular nucleotide effects on HCC cell proliferation, two independent HCC patient cohorts were analyzed for P2 purinergic receptor expression, and nucleotide treated HCC cell lines were evaluated for effects on proliferation and cell cycle progression. Our studies suggest that multiple P2 purinergic receptor isoforms are overexpressed in liver tumors, as compared to uninvolved liver, and dysregulation of P2 purinergic receptor expression is apparent in HCC cell lines, as compared to human primary hepatocytes. High P2X3 purinergic receptor expression is associated with poor recurrence-free survival (RFS), while high P2Y13 expression is associated with improved RFS. Extracellular nucleotide treatment alone is sufficient to induce cell cycle progression, via activation of JNK signaling, and extracellular ATP-mediated activation of P2X3 receptors promotes proliferation in HCC cells. Conclusion: Our analysis of HCC patient livers and HCC cells *in vitro* identifies a novel role for dysregulation of P2 purinergic signaling in the induction of hyper-proliferative HCC phenotype and identifies P2X3 purinergic receptors as potential new targets for therapy.

## INTRODUCTION

Hepatocellular carcinoma (HCC) is the second leading cause of cancer deaths worldwide [1]. The incidence and deaths resulting from HCC have steadily increased in the US over the past three decades, and it is predicted that they will continue to rise [2]. Currently, the prognosis for HCC is dismal with overall survival rates of 3-7% [3, 4]. Treatment options for HCC are limited, with liver resection or orthotopic liver transplantation (OLT) as the best approaches. Resection has a very high recurrence rate of up to 70% at 5 years. OLT has a better prognosis, however most patients are not suitable candidates and a scarcity of organs contribute to longer wait times; more than 16, 000 patients were on the wait list for livers in 2012 [5, 6]. Over 75% of HCC are caused by viral infections such as Hepatitis C Virus (HCV) and 70-90% of all tumor development is associated with chronic liver injury, inflammation and cirrhosis [3].

It is well established that liver injury with cellular stress and inflammation is a potent trigger for ATP release from hepatocytes and other liver cells [7-9]. Extracellular ATP *via* the activation of cell surface P2 purinergic receptors influences cell proliferation, differentiation and apoptosis [10]. We have previously shown that extracellular ATP-mediated P2 purinergic receptor activation promotes cell-cycle progression and proliferation in rat primary hepatocytes *via* c-Jun N-terminal Kinase (JNK) pathway *in vitro* and hepatocyte proliferation in response to 70% partial hepatectomy *in vivo* [11, 12]. Extracellular ATP-mediated activation of P2X (ligand gated ion channels) and P2Y (G protein-coupled) receptors have been reported to influence cell proliferation, migration or apoptosis of various cancer cell types [10, 13-16]. Studies suggest that extracellular ATP-mediated activation of P2Y2 receptor promotes proliferation and migration in HCC cells[17]; however the role of the remaining 14 P2 receptor isoforms in HCC is currently unknown. ATP levels in the tumor interstitium of mice was measured in the hundreds micro molar range compared to near undetectable levels in healthy tissues [18].

Ectonucleotidases such as CD39 decrease extracellular nucleotide concentrations by hydrolyzing nucleotides to nucleosides and ultimately adenosine [19, 20]. Deletion of *Cd39* in mice is shown to increase hepatocyte proliferation and promote hepatocarcinogenesis [20]. Furthermore, P2Y2 mRNA and protein expression are increased in human HCC cells compared to normal hepatocytes and others have shown that there is increased P2Y2 and P2Y4 receptor expression in other cancers [17, 21, 22]. Recently, peritumoral P2X7 purinergic receptor expression has been associated with poor survival in HCC patients after surgical resection[23]. However, P2 purinergic receptor expression and its role in hepatocyte cell cycle progression in human HCC remain unexplored.

The purpose of this study was to examine the role of P2 purinergic signaling in the pathogenesis of HCC in patients and characterize the influence of extracellular nucleotides on HCC cell proliferation. Our analysis reveals dysregulation of P2 purinergic receptor expression in HCC tumors, as compared to the uninvolved area of the same patient, and compared to normal livers. Increased frequency of P2 purinergic receptor upregulation in patients with HCV *versus* those with non-viral etiologies identifies a unique subset of viral-induced HCC overexpressing P2 purinergic receptors. We show that P2X3 purinergic receptor overexpression is associated with poor recurrence-free survival in patients with HCC. Our *in vitro* findings suggest that nucleotide treatment alone was sufficient to induce HCC cell proliferation, and provide mechanistic insights into the potential role of dysregulation of purinergic signaling in the induction of hepatocyte cell-cycle control associated with HCC pathogenesis.

## RESULTS

### Increased P2 purinergic receptor mRNA expression in Human HCC livers

**Figure 1:**
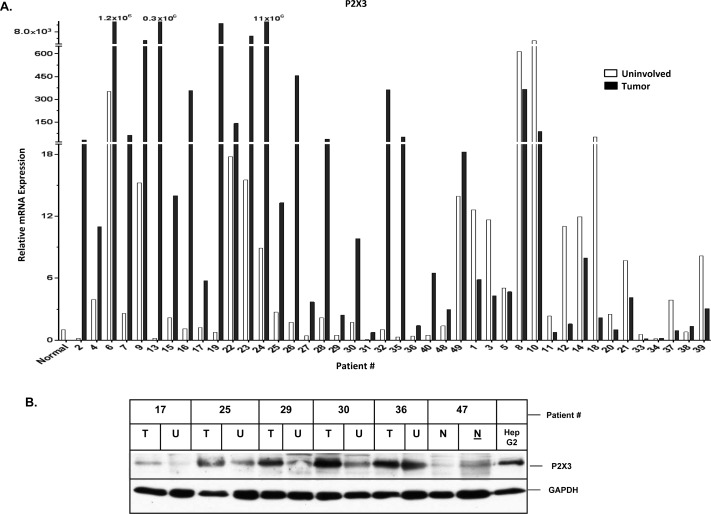

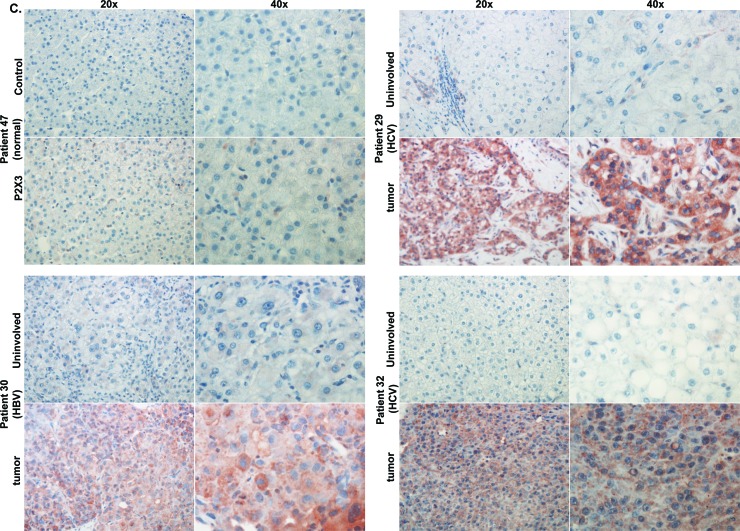

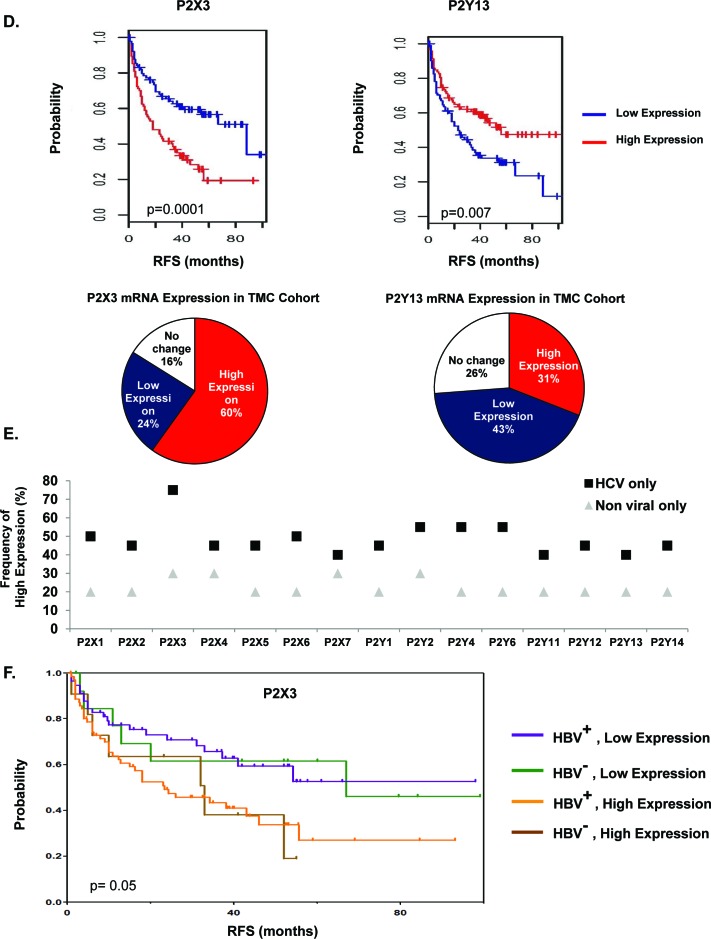
Increased P2X3 purinergic receptor mRNA expression is associated with poor recurrence-free survival in HCC patients **A.** RNA isolated from HCC tumors, adjacent uninvolved areas and normal livers were analyzed by qRT-PCR. Relative mRNA expression was calculated with reference to normal livers. **B.** Western Blotting of total proteins isolated from TMC cohort patient tumor (T, 20 μg), uninvolved areas (U, 20 μg), normal liver tissue (N, 20 & 45 μg) and HepG2 cells (20 μg). **C.** Immunohistochemical analysis of P2X3 expression in normal human liver and TMC cohort liver tumors. **D.** Recurrence-free survival analysis (Kaplan-Meier) of Korean patient cohort; ‘high’ (above median) *vs* ‘low’ (below median) expression of P2X3 and P2Y13 in HCC tumors, *n* = 188. Distribution of P2X3 and PY13 mRNA expression in TMC patient cohort, *n* = 42. **E.** Frequency of ‘high’ (≥ 2-fold) P2 purinergic receptor mRNA expression, as compared to uninvolved areas in HCC tumors of patients with HCV *vs*. non-viral etiologies. **F.** Recurrence-free survival analysis (Kaplan-Meier) of Korean patient cohort; ‘low’ P2X3 (below median) and HBV positive *vs* ‘low’ P2X3 (below median) and HBV negative *vs* ‘high P2X3 (above median) and HBV positive *vs* ‘high P2X3 (above median) and HBV negative in HCC tumors, *n* = 156.

To determine if P2 purinergic receptor expression is dysregulated in HCC livers, we analyzed 42 pairs of HCC livers (uninvolved *vs*. tumor) and 6 normal donor liver samples from the TMC cohort. Twenty-one patients were infected with Hepatitis C Virus (HCV), 5 patients with Hepatitis B Virus (HBV) and 10 patients with non-viral etiologies. Information on the etiology was unavailable for 6 patients (Suppl. Table 1). Relative expression of all 15 P2 purinergic receptor isoforms was analyzed by qRT-PCR. Multiple P2 purinergic receptor isoforms were elevated ≥ 2-fold in liver tumors (‘high’ expression) as compared to uninvolved areas of the liver (Table 1). Our results reveal that 31% of patients in the TMC cohort exhibited ‘high’ expression of at least one P2 purinergic receptor isoform, while 60% of patients exhibited ‘high’ P2X3 mRNA expression in liver tumors, compared to their uninvolved areas (Figure 1A). P2X3 protein overexpression was observed in HCC tumors compared to uninvolved livers by Western blotting of total homogenates (Figure 1B). HepG2 cell total protein was used as a positive control. Further validation was done by immunohistochemical analysis of tumors, uninvolved areas of HCC patient livers, and normal liver which revealed that P2X3 protein overexpression was predominant in hepatocytes (Figure 1C). P2 purinergic receptor mRNA expression was significantly elevated in HCC tumors, as compared to uninvolved areas; despite apparent dysregulation of P2 purinergic receptor expression in the uninvolved areas of most of the patient livers, as compared to normal livers (Table 1). These findings suggest that dysregulation of P2 purinergic receptor expression in chronic liver injury may precede HCC development.

**Table 1:** Purinergic receptor expression in HCC patients in the TMC Cohort

Receptor Subtype	High Expression (%)	High Expression (%)
	(vs uninvolved)	(vs normal)
P2X3	60	79
P2Y14	48	67
P2Y2	43	40
P2Y6	43	79
P2X6	40	52
P2Y4	40	77
P2X1	36	52
P2X2	36	83
P2X4	36	38
P2X5	36	60
P2X7	33	67
P2Y1	33	43
P2Y11	33	55
P2Y12	33	43
P2Y13	31	40

Frequency (%) of HCC patients with high mRNA expression of P2 Purinergic receptor subtypes as compared the uninvolved liver or normal liver.

### P2 purinergic receptor expression correlates with HCC patient survival

The upregulation of multiple P2 purinergic receptor isoforms observed in HCC prompted us to question the significance of dysregulation of P2 purinergic receptor expression on HCC survival in a larger Korean cohort (188 patients). Demographic and pathologic features of this Korean cohort are presented in Suppl. Table 2. Patients were stratified according to ‘high’ (above median) and ‘low’ (below median) P2 purinergic receptor expression based on microarray gene expression analysis of resected livers. Patients with ‘high’ P2X3 expression had a significantly lower recurrence-free survival (RFS) rate than those patients with ‘low’ P2X3 receptor expression (*p* = 0.0001). On the other hand, patients with high P2Y13 expression had significantly improved recurrence free survival (*p* = 0.007) (Figure 1D). Recall that in the TMC cohort P2X3 was observed as the receptor with the greatest frequency of ‘high’ expression in HCC tumor samples (60%) and P2Y13 was identified as the receptor with the lowest frequency of ‘high’ expression (31%) (Figure 1D).

Corroborating our findings, Oncomine analysis revealed that P2X3 mRNA is significantly overexpressed in the Mas_Liver dataset (*p* = 9.23E-7), while P2Y13 mRNA is underexpressed in the Chen Liver dataset (p = 1.03E-14) (Suppl. Figure 1) [25, 26]. It is noteworthy that P2X3 was ranked among the top 7% overexpressed genes in the Mas_Liver dataset, which included exclusively HCV-positive HCC samples and HCV-negative normal livers. In the TMC cohort 43% of patients exhibited ‘low’ P2Y13 mRNA expression, which was ranked among the top 4% underexpressed genes in Chen_Liver dataset, and included HCC samples of viral and non-viral origin. Dysregulation of P2X3 and P2Y13 mRNA expression was evident in HCC livers, despite comparable DNA copy numbers of these genes between HCC and normal in the TCGA, Guichard_Liver and Guichard_Liver 2 DNA datasets [24, 29].

### P2 purinergic receptor upregulation is more frequent in HCV patients

In order to gain insight into the etiology of HCC and its influence on P2 purinergic receptor overexpression in HCC livers, we stratified our TMC patients based on their history of viral infection and found that P2 purinergic receptor upregulation, as assessed by qRT-PCR was more prevalent among those patients infected with HCV, as compared to non-viral groups (Figure 1E). P2X3 overexpression is evident in 75% of HCV patients as compared to only 30% of non-viral patients. Immunohistochemical analysis of patient livers with HBV (patient # 30), HCV (patient # 29, 32, 36) and non viral patients (patient # 16, 28, 50) indicates that P2X3 overexpression in HCC tumors as compared to uninvolved livers can be detected in HCC livers, irrespective of etiology (Figure 1C & Suppl. Figure 2).

To determine whether viral infection influences recurrence free survival, Kaplan-Maier analysis was done on the Korean cohort. Patients were stratified as HBV positive or negative AND P2X3 ‘high’ or ‘low’ as previously defined. Regardless of HBV status, patients with ‘high’ P2X3 expression had lower RFS than patients with ‘low’ P2X3 expression (Figure 1F). Among HBV positive patients, those with high P2X3 expression had significantly lower RFS compared to those with low P2X3 expression (Suppl. Figure 3A). While there was a considerable difference in median survival between P2X3 high (33 months) and P2X3 low (67 months) among HBV viral patients, there was no statistically significant difference in RFS between the two, likely due to small sample size (25 patients) (Suppl. Figure 3B). To determine whether the impact of P2X3 overexpression on risk of recurrence was dependent of HBV status Cox proportional hazard model analysis was performed. Our data reveals that there was no significant interaction between P2X3 expression and HBV status (*p* = 0.90), confirming that the effect of P2X3 high expression on the risk of recurrence does not significantly differ across HBV groups.

### Dysregulation of P2 purinergic receptor expression in HCC cells

**Figure 2:**
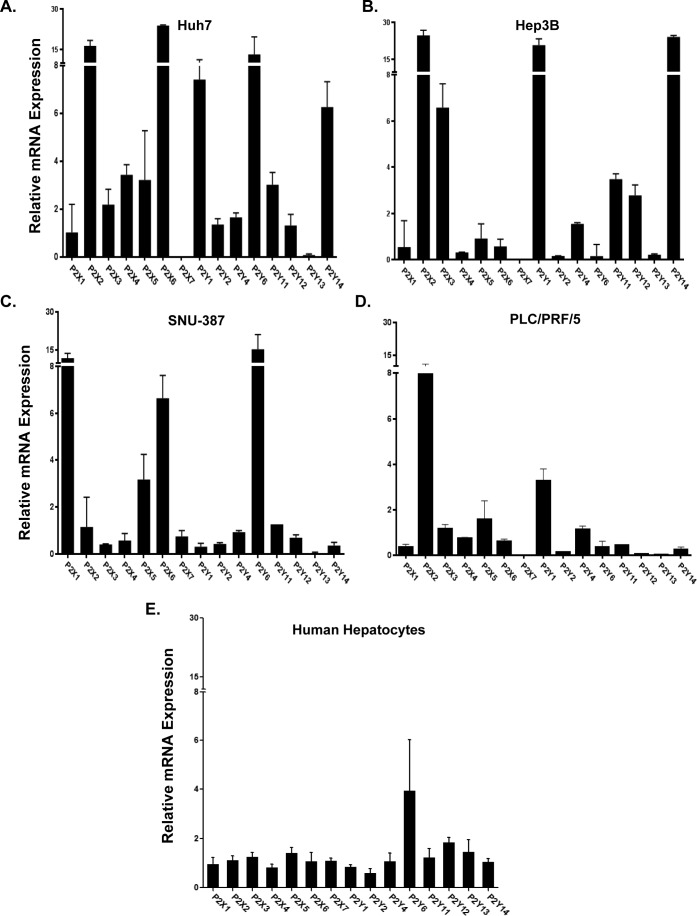
Dysregulation of P2 purinergic receptor expression in HCC cell lines RNA isolated from **A.** Huh7, **B.** Hep3B, **C.** SNU-387, **D.** PLC/PRF/5 was analyzed by qRT-PCR for mRNA expression and relative expression was calculated with reference to primary hepatocytes isolated from normal healthy adults (*n* = 4). **E.** Total RNA isolated from freshly-frozen primary human hepatocytes (healthy adults with no known HCC; *n* = 4) were analyzed by qRT-PCR for all 15 isoforms of P2 purinergic receptors. Purinergic receptor mRNA expression was calculated with reference to GAPDH, housekeeping gene.

To determine if dysregulation of P2 purinergic receptor expression is associated with hepatocyte transformation to HCC phenotype and to identify a suitable *in vitro* model system for mechanistic studies, we analyzed P2 purinergic receptor mRNA expression by qRT-PCR in four human hepatocellular carcinoma cell lines - Huh7, Hep3B, SNU-387, PLC/PRF/5, and normal human primary hepatocytes. Suggesting a role for P2 purinergic receptors in hepatocyte transformation, all four HCC cell lines had upregulation of multiple P2 purinergic receptor isoforms and lower expression of P2Y13. Huh7, Hep3B and PLC/PRF/5 had lower expression of P2X7 and upregulation of P2X2 (Figure 2A-2D). Relative mRNA expression of all 15 purinergic receptor isoforms was comparable in normal human primary hepatocytes (Figure 2E).

### Extracellular nucleotides induce proliferation and cell-cycle progression in HCC cells

**Figure 3:**
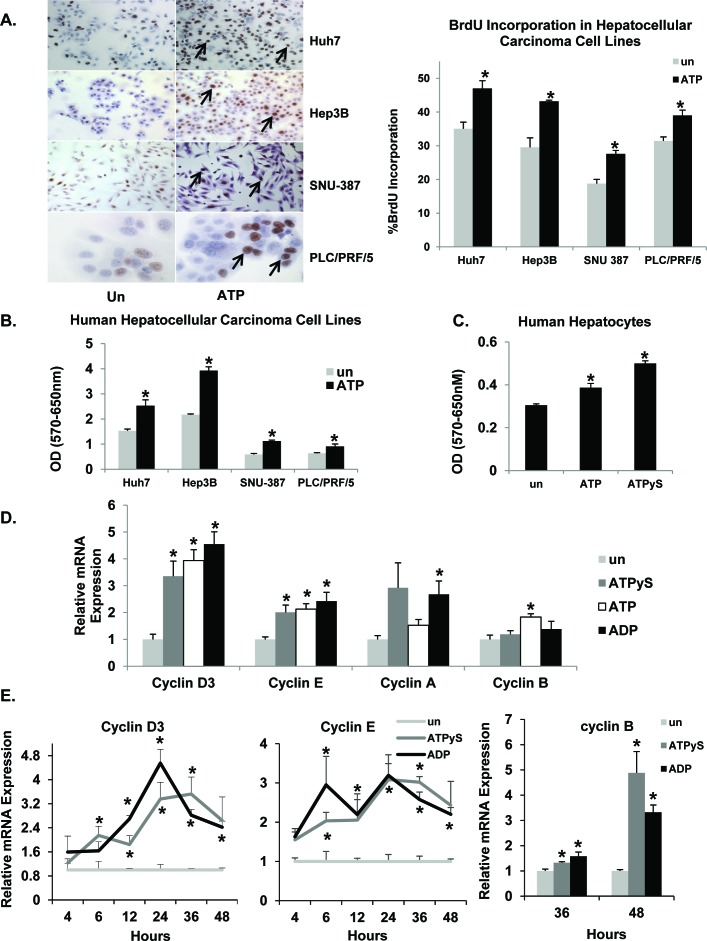

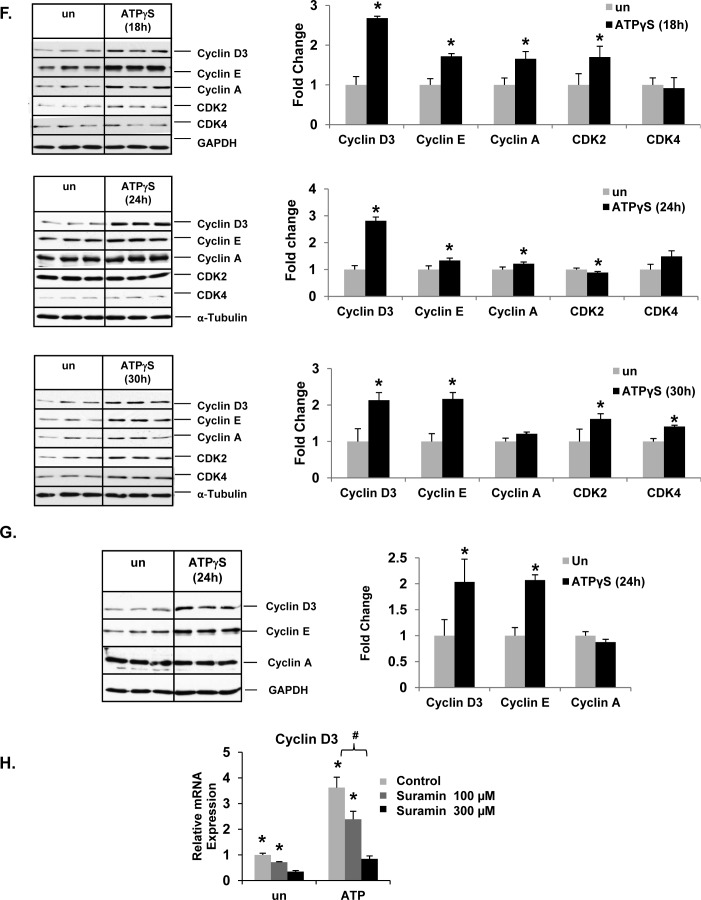
Extracellular nucleotides induce cell-cycle progression and proliferation in liver cancer cell lines **A.** HCC cells after 24h of ATP treatment. Light microscopic images (Huh7, Hep3B, SNU-387-10X; PLC/PRF/5 – 40x) of BrdU immunostained cells, expressed as a percentage of total cells. Arrows point to nuclei with BrdU staining. **B.** HCC cell proliferation after 18h ATP treatment, assessed by MTT assay. **C.** Human hepatocyte cell proliferation after 18h ATP or ATPγS treatment, assessed by MTT assay. **D.** Total RNA isolated from Huh7 cells treated with ATPγS, ATP or ADP for 24h and analyzed by quantitative RT-PCR for cyclin mRNA. **E.** Temporal expression of cyclin mRNA. **F.** Western blotting of total proteins from Huh7 cells after 18, 24 or 30h ATPγS treatment. **G.** Western blotting of total proteins from human hepatocytes after 24h ATPγS treatment. **H.** Cyclin mRNA expression in Huh7 cells after 24h of nucleotide treatment ± pre-treatment (30 min) with suramin. Data represented as the mean ± SEM, *n* = 3, *p < 0.05 *vs*. untreated, ^#^*p* < 0.05.

To determine whether P2 purinergic receptor activation influences HCC cell proliferation, BrdU incorporation was assessed in HCC cells maintained in SFM for 24h and treated with ATP for 24h. ATP treatment alone was sufficient to induce proliferation in each of these cell lines (Huh7, 34%; Hep3B, 46%; SNU-387, 47% and PLC/PRF/5, 24%), identifying nucleotides as liver cancer cell mitogens (Figure 3A). Extracellular ATP can be hydrolyzed to ADP and adenosine by ectonucleotidases at the plasma membrane. Therefore, we tested the effects of ATPγS (non-hydrolyzable analog of ATP) or ADP (break down product of ATP) (100 μM) on Huh7 cell proliferation. ATPγS treatment alone was sufficient to induce BrdU incorporation as early as 12h (42%); maintained at 18h (30%) and 24h (29%) in Huh7 cells (Suppl. Figure 4). ADP was also able to independently increase the number of BrdU positive nuclei (12h, 30%; 18h, 35%; 24h, 37%) (Suppl. Figure 4). Furthermore, ATP treatment (18 h) increased cell proliferation and viability in all four cell lines as determined by MTT assay (Huh7, 65%; Hep3B, 81%; SNU-387, 90% and PLC/PRF/5, 43%) (Figure 3B). In human hepatocytes, ATP treatment (18h) led to a modest increase in cell proliferation and viability (27%) while ATPγS treatment (18h) was more potent (64%) (Figure 3C).

Nucleotide treatment resulted in increased cyclin D3, cyclin E, cyclin A and cyclin B mRNA expression, correlating with increased proliferation (Figure 3D). Cyclin D3 mRNA expression was observed after 6h (ATPγS - 2.1 fold), 12h (ATPγS - 1.8 fold; ADP - 2.7 fold), 24h (ATPγS - 3.4 fold; ADP - 4.5 fold), 36h (ATPγS - 3.5 fold; ADP - 2.8 fold) and 48h (ADP - 2.4 fold) treatment, as compared to untreated cells (Figure 3E). Cyclin E expression exhibited a similar profile while cyclin B expression was induced later at 36h and 48h after treatment (Figure 3E). Western blotting of Huh7 cell total lysates revealed that ATPγS treatment (18h, 24h, 30h) alone was sufficient to induce cyclin D3 (2.7, 2.8, 2.1 fold), cyclin E (1.7, 1.3, 2.2 fold) and cyclin A (1.7, 1.2, 1.2 fold) protein expression (Figure 3F). ATPγS treatment (30h) induced CDK2 (1.6 fold) and CDK4 (1.4 fold) protein expression. Additionally, ATP treatment alone was sufficient to induce cyclin D3 protein expression in three of the four HCC cell lines tested; Huh7 (3.2 fold), SNU-387 (1.6 fold) and PLC/PRF/5 (1.4 fold)) (Suppl. Figure 5). ATPγS treatment (24h) of human hepatocytes induced cyclin D3 (2.0 fold) and cyclin E (2.0 fold) protein expression (Figure 3G). These data suggest that nucleotide treatment alone was sufficient to promote cell cycle progression and proliferation in human hepatocytes as well as liver cancer cells.

To confirm that nucleotide effects on dysregulation of cyclin expression is mediated *via* the activation of P2 purinergic receptors, cells were treated with a broad spectrum P2 purinergic receptor antagonist - suramin, prior to nucleotide treatment. Suramin pre-treatment resulted in a dose dependent attenuation of cyclin D3 mRNA induction (Figure 3H). Suramin treatment alone reduced baseline cyclin D3 expression (Figure 3H), suggesting a role for endogenous ATP release and extracellular nucleotide signaling in basal Huh7 cell cycle progression.

### Nucleotides induce cyclin expression *via* c-Jun N-terminal kinase (JNK) signaling in Huh7 cells

**Figure 4:**
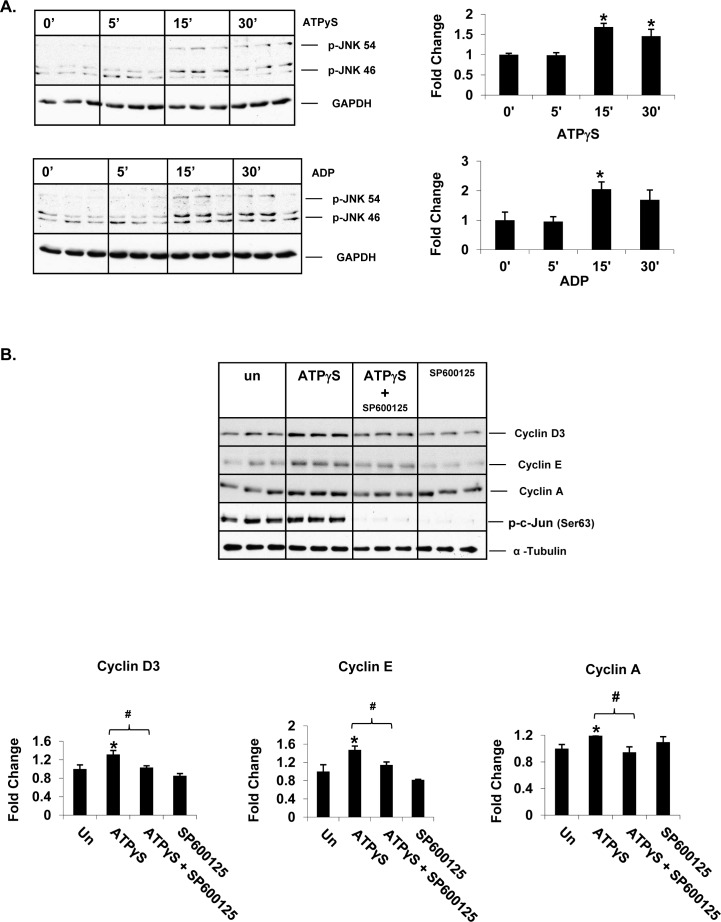
Extracellular nucleotides induce cell-cycle progression via activation of JNK signaling in Huh7 cells Western Blotting of total protein extracts of Huh7 cells after **A.** 5, 15 and 30min of ATPγS or ADP treatment (100 μM), **B.** ATPγS (24 hr) ± pre-treatment (30 min) of JNK inhibitor, SP600125 (30 μM).

We have previously shown that extracellular nucleotide-mediated activation of JNK signaling induces cell-cycle progression and proliferation of rat primary hepatocytes *in vitro* [11]. To determine whether extracellular nucleotides induce JNK signaling in transformed hepatocytes, Huh7 cells were treated with nucleotides for 5, 15 and 30 min and total protein extracts were analyzed by Western blotting for JNK phosphorylation (Ser ^Thr/Tyr^). ATPγS treatment alone was sufficient to induce JNK phosphorylation as early as 15 min (1.7 fold) (Figure 4A). ADP treatment resulted in similar induction (15 min - 2 fold).

To determine if extracellular nucleotide-mediated activation of JNK signaling influences cell-cycle progression in transformed hepatocytes, Huh7 cells were treated with SP600125 (JNK inhibitor) prior to ATPγS treatment. Efficiency of JNK inhibition in Huh7 cells was confirmed by Western blotting for p-c-Jun. JNK inhibition completely attenuated ATPγS-mediated induction of cyclin D3, E and A proteins (Figure 4B) identifying a role for the activation of JNK signaling pathway in P2 purinergic receptor-mediated induced cell-cycle progression in Huh7 cells.

### P2X3 antagonism attenuates nucleotide-induced proliferation and cell cycle progression

**Figure 5:**
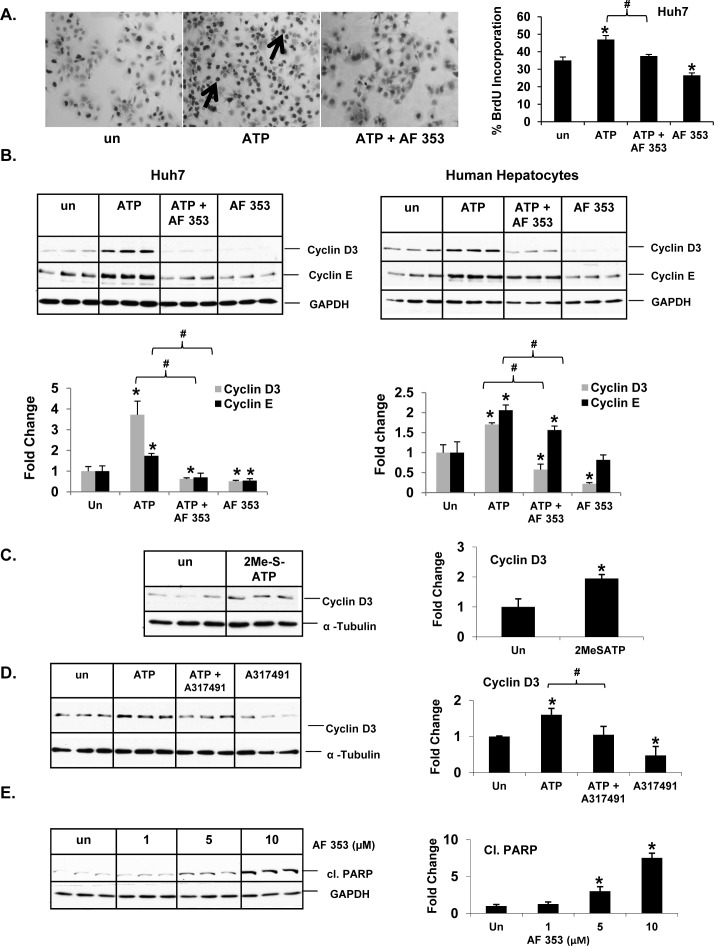
P2X3 antagonist, AF-353, attenuates ATP-mediated induction of Huh7 cell proliferation **A.** Light microscopic images (10X) of BrdU immunostained Huh7 cells after ATP (100 μM, 24 hr) ± pre-treatment with AF- 353 (5 μM). Arrows point to BrdU-positive cells, expressed as a percentage of total cells. Data represents mean ± SEM, *n* = 3-6, *p < 0.05 *vs*. untreated. Western Blotting of total proteins extracted from Huh7 cells and human hepatocytes treated (24 h) with **B.** ATP ± P2X3 antagonist, AF- 353 (5μM), **C.** P2X3 agonist, 2MeSATP (50μM), **D.** ATP ± A317491 (30μM), **E**. P2X3 antagonist, AF- 353 alone. Data represents mean ± SD, *n* = 3, *p < 0.05 *vs*. untreated, ^#^*p* < 0.05.

Prompted by our identification of P2X3 as the most frequently overexpressed purinergic receptor isoform in human HCC tumors in our TMC cohort and that P2X3 overexpression is associated with poor recurrence-free survival in our Korean cohort, we performed a series of mechanistic studies in Huh7 cells *in vitro,* to determine the functional significance of P2X3 and its role in nucleotide-induced proliferation. Huh7 cells were treated with a highly selective P2X3 antagonist AF-353 prior to ATP treatment. Implicating P2X3 receptors in nucleotide-mediated proliferation, AF-353 pre-treatment completely abolished ATP-mediated increase in BrdU incorporation in Huh7 cells (Figure 5A). Similarly, ATP-mediated induction of BrdU incorporation in Hep3B cells was completely attenuated by pre-treatment with P2X3 antagonist (Suppl. Figure 6). ATP-mediated induction of cyclin D3 and cyclin E expression were completely attenuated in Huh7 cells and human hepatocytes pre-treated with AF-353 (Figure 5B). Our observations that P2X3 agonist 2-MeSATP treatment alone was sufficient to induce cyclin D3 expression (1.9 fold) and pre-treatment with an alternative P2X3 antagonist, A317491 attenuated ATP induced cyclin D3 expression in Huh7 cells further validates the role of P2X3 purinergic receptor activation in the induction of cell-cycle progression in HCC cells (Figure 5C & 5D) [32-34].

Interestingly, P2X3 antagonist treatment alone was sufficient to reduce the baseline BrdU-positive cells and cyclin protein expression in serum-free conditions, implicating P2X3 activation as a necessary facilitator of Huh7 cell cycle progression (Figure 5A-5B). Additionally, P2X3 antagonist treatment alone resulted in increased cleaved PARP protein expression, an established marker of cells undergoing apoptosis (Figure 5E).

### P2X3 overexpression increases basal and ATP mediated cell proliferation

**Figure 6:**
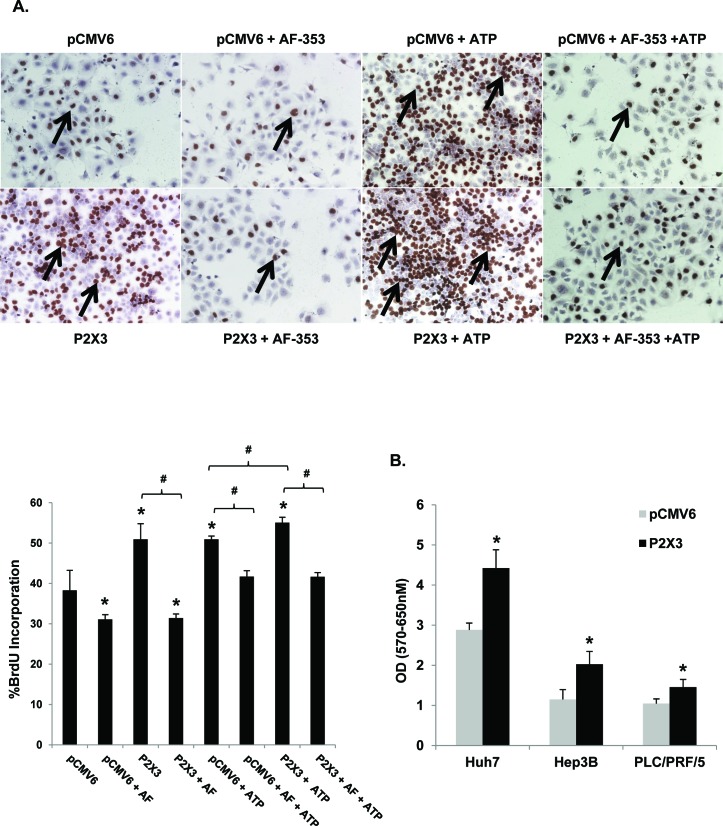
P2X3 overexpression induces basal and ATP mediated proliferation **A.** Light microscopic images (10X) of BrdU immunostained Huh7 cells after pCMV6 plasmid or P2X3 DNA transfection ± ATP (100 μM, 24 hr) ± pre-treatment with AF- 353 (5 μM). Arrows point to BrdU-positive cells, expressed as a percentage of total cells. **B.** HCC cell proliferation after 72h pCMV6 plasmid or P2X3 DNA transfection, assessed by MTT assay. Data represents mean ± SEM, *n* = 3-6, *p < 0.05 *vs*. untreated.

To confirm P2X3 receptor involvement in HCC cell proliferation, P2X3 was overexpressed in Huh7 cells then these cells were treated with ATP +/− P2X3 antagonist, AF-353. BrdU analysis revealed that P2X3 overexpression (72h post-transfection) alone was sufficient to significantly increase proliferation (51%) compared the vector control pCMV6 (38%). AF-353 treatment (24h) completely attenuated the P2X3 mediated increase in proliferation (Figure 6A). ATP Treatment (24h) further increased BrdU incorporation when compared to the pCMV6 control. ATP mediated proliferation was significantly higher in cells overexpressed with P2X3 compared to those transfected with only pCMV6. Again, pre-treatment with AF-353 significantly attenuated the ATP mediated increase in BrdU incorporation. Furthermore, P2X3 overexpression alone was sufficient to improve cell proliferation and viability of Huh7, Hep3B and PLC/PRF/5 cells as assessed by MTT assay (Figure 6B)

Collectively, these findings suggest that P2X3 purinergic receptor expression and function is critical for HCC cell survival and basal proliferation as well as proliferation in response to changes in nucleotide concentrations in the extracellular milieu.

## DISCUSSION

In this study, we undertook a comprehensive analysis of all 15 P2 purinergic receptor isoforms in HCC tumors, as compared to the adjacent uninvolved areas of HCC livers as well as normal livers. Our studies have identified a distinct dysregulation of P2 purinergic receptor expression in HCC livers. The upregulation of P2 purinergic receptor expression observed among the tumor samples when compared to their adjacent uninvolved areas highlights its significance in the pathogenesis of HCC. Altered P2 purinergic receptor expression in the uninvolved areas of HCC livers (as compared to normal healthy livers) suggests that purinergic signaling may be dysregulated prior to HCC development. Therefore, as potential biomarkers, P2 purinergic receptor upregulation has the advantage of being detected prior to tumor development when effective interventions may be undertaken. Another significant finding of this study is that there is a higher frequency of P2 purinergic receptor upregulation in HCC tumors of HCV patients, as compared to those tumors with non-viral etiologies, identifying a specific subgroup of HCC with higher prevalence of P2 purinergic receptor overexpression. However, immunohistochemical analysis suggests that P2X3 overexpression is evident in HCC tumors, irrespective of viral status. Further studies in larger patient cohorts are required to determine the impact of viral infection on the dysregulation of P2 purinergic receptor expression and its role in HCC pathogenesis.

Dysregulation of purinergic receptor expression may influence initiation and temporal progression of HCC *via* its influence on dysregulation of cell-cycle control, a hallmark of HCC cells. In this study, we have identified that P2X3 purinergic receptor expression and function is critical for HCC cell survival and proliferation. Interestingly, P2X3 was the most frequently upregulated purinergic receptor isoform in our local TMC patient cohort. It was ranked in the top 7% of overexpressed genes in the Mas_Liver dataset (Oncomine) and its ‘high’ expression had the strongest correlation with decreased recurrence free survival in our Korean patient cohort [26, 30]. Therefore, P2 purinergic receptors can serve as potential new targets for the development of HCC therapeutics.

While purinergic signaling has been implicated in cancer, its influence on proliferation varies by cancer cell type and cell-type specific P2 purinergic receptor expression profile [10, 13, 14, 17-22]. We observed differential P2 purinergic receptor expression profiles among the four HCC cell lines tested (Figure 2). This heterogeneity of purinergic receptor expression profile may dictate varying functional outcomes in cells, in response to nucleotide changes in the extracellular milieu. Activation of each receptor isoform elicits unique responses, such that a cancer cell may benefit from increased expression of some isoforms and decreased expression of others. Confirming previous findings, P2X7, and P2Y13 expression were reduced, as compared to the other receptor isoforms, in three of the four HCC cell lines tested [35, 36]. P2X7 purinergic receptor activation induces apoptosis by increasing intracellular levels of Ca^2+^ in multiple cell types [10]. It is no surprise then that this receptor is down regulated in cancer cells as a means of avoiding cell death. In our TMC patient cohort, P2X7 had a lower frequency of upregulation (33%), as did P2Y13 (31%), as compared to other receptors such as P2X3 (60%) (Table 1). Our data show a strong positive correlation between ‘low’ P2Y13 expression and increased recurrence free survival in our Korean cohort (Figure 1D). P2Y13 purinergic receptor, preferentially activated by ADP, has been shown to play a role in the activation of anti-oxidant Nrf2/HO-1 axis to protect against oxidative stress-induced neuronal death [37]. It is well established that oxidative stress contributes to the development of HCC and it is reported that patients with HCC that exhibit increased oxidative stress levels are prone to recurrence after ‘curative’ treatment [38, 39]. P2X2 expression was elevated in three HCC cell lines tested. Compared to the normal livers, P2X2 had the highest frequency of upregulation in HCC tumor tissues (83%) (Table 1). It is noteworthy that P2X2 often forms heteromultimeric complexes with P2X3, the other highly overexpressed receptor in our TMC patient cohort [40]. Overall, P2 purinergic receptor expression is dysregulated in HCC cells commensurate with its hyper-proliferative transformed phenotype, as compared to quiescent hepatocytes isolated from normal healthy adult livers.

Our finding that extracellular nucleotides induce cyclin D3, cyclin E and cyclin A protein expression *via* activation of JNK signaling is of significant interest to our understanding of pathogenesis of HCC, as it has been previously reported that JNK1 expression is increased in primary hepatocellular carcinomas [41, 42]. Cyclin D3 was reported to be upregulated in 51-72% of HCC tissues and was overexpressed in the Mas_liver dataset (Supp. Figure 7) [26, 43, 44]. Our *in vitro* studies suggest nucleotide induced cyclin D3 expression is mediated *via* activation of P2X3 purinergic receptors in Huh7 cells. We show that nucleotide treatment induces CDK4 protein expression. Studies suggest that the function of CDK4 is most critical for G_1_/_S_ HCC cell cycle progression and that CDK4 is activated by cyclins D and E [45, 46].

P2X3 overexpression increases cell proliferation and viability, indicating for the first time the critical role of P2X3 receptors in HCC cell growth. These findings highlight the functional significance of increased P2X3 receptor expression in the tumors. Furthermore, the attenuation of P2X3 mediated proliferation by P2X3 antagonist, AF-353, clearly highlights the potential therapeutic application by pharmaceutical control of P2X3 receptor activation against HCC propagation.

P2X3 receptor expression in afferent neurons and its role in pain sensation is well characterized [33]. Although P2X3 expression has been reported in hepatocytes, cholangiocytes, and portal vein myocyctes in the liver and liver derived cell lines, P2X3 receptor function in hepatic cells has not been well characterized [36, 47-49]. Recently, P2X3 has been reported to be involved in the complex regulation of liver regeneration through its expression on Natural Killer (NK) cells [50, 51]. It is reported that NK cells are reduced in the intratumoral tissue of HCC patients, particularly those with advanced stage HCC [52, 53]. In the present study, we provide evidence for P2X3 overexpression in hepatocytes, as assessed by immunohistochemical analysis of HCC patient livers and its role in hepatocyte proliferation, as assessed by *in vitro* studies in human primary hepatocytes and four independent HCC-derived cell lines. Further studies are required to determine if P2X3 purinergic receptor expression in immune cells infiltrating HCC tumors plays a role in HCC pathogenesis.

Considering previous findings of increased ATP concentrations in the extracellular milieu in injured and inflamed livers [8] and in the tumor interstitium [18], as well as our findings of increased P2 purinergic receptor expression in HCC liver tumors, our observations of nucleotide-mediated increased proliferation in HCC cells is particularly meaningful.

In conclusion, extracellular nucleotides *via* the activation of P2 purinergic receptors induce proliferation and cell cycle progression in HCC cells. P2 purinergic receptor expression is significantly dysregulated in HCC tumor tissues and exhibits strong correlation with recurrence-free survival in HCC patients. These studies underscore the potential role of purinergic signaling in the pathogenesis of HCC, and highlight the utility of P2 purinergic receptors as a potential new class of biomarkers and therapeutic targets.

## MATERIALS AND METHODS

### HCC patients

Liver tumors and adjacent, uninvolved areas (42 pairs) were obtained from HCC patients undergoing resection or liver transplantation at St. Luke's Episcopal Hospital and Ben Taub Hospital in the Texas Medical Center, Houston TX (TMC cohort). Normal livers (6 samples) were obtained from donor livers prior to transplantation at the St. Luke's Episcopal Hospital.

Tumor specimens were obtained from an additional 188 HCC patients undergoing hepatectomy at Seoul National University, Guro Hospital of Korea University, Seoul, Chonbuk National University, Jeonju, and Dong-San Medical Center of Keimyung University, Daegu, Korea (Korean cohort). Gene expression data from the Korean cohort were generated using the Illumina microarray platform human-6 versions 2 and 4 (Illumina, San Diego, CA). These patients were followed up prospectively at least once every 3 months after surgery. Primary microarray data from the Korean cohort are available in NCBI's GEO public database (accession numbers GSE16757 and GSE43619). The study protocols were approved by the Institutional Review Boards of institutions, and all participants had provided written, informed consent.

### Oncomine database

The Oncomine 4.5 (www.oncomine.org), a publicly available database of published cancer gene expression profiles, was queried for alterations in P2 purinergic receptor (P2X3, P2Y13) and cyclin D3 genes with additional filters defined for the analysis type (cancer *vs* normal) and cancer type: liver cancer. Five gene expression and three DNA copy number datasets were retrieved for further analysis, comparing HCC *vs* normal [24-29]. All gene expression data were log-transformed and median-centered and all statistical analyses were performed using functions implemented in Oncomine. P value of less than 0.05 (*p* < 0.05) is considered significant [30].

### Hepatocytes, HCC cell lines and gene transfection

Normal human primary hepatocytes isolated from healthy adults (no known history of HCC), in supsension or freshly frozen prior to shipment (Cryoport Systems, CA) were purchased from Triangle Research Labs, NC. Human hepatocellular carcinoma derived Huh7, Hep3B, SNU-387 and PLC/PRF/5, cell lines were maintained, as described in the Supplementary Methods.

### Immunohistochemistry, MTT assay and western blotting

Formalin-fixed and paraffin embedded liver sections from HCC patients were analyzed by immunohistochemistry. HCC cell proliferation was evaluated by immunohistochemical analysis of BrdU incorporation and MTT assay; Total protein extracts of HCC cells were analyzed by Western blotting, as described in Supplementary Methods.

### Real-time quantitative reverse-transcriptase polymerase chain reaction (qRT-PCR)

Total RNA was isolated from human livers or cells using Trizol Reagent according to manufacturer's instructions (Invitrogen, NY). Complementary DNA (cDNA) synthesis and qRT-PCR were performed, as described in Supplementary Methods.

### Statistical analysis

Data was analyzed by one way analysis of variance (ANOVA) or unpaired Student's *t* test. Values of *p* < 0.05 were considered statistically significant. TMC cohort patients were stratified by P2 purinergic receptor expression (‘high’ - ≥ 2-fold *vs* ‘low’ - ≤ 0. 5 fold as compared to uninvolved areas). Korean cohort patients were stratified according to ‘high’ (above median) and ‘low’ (below median) expression for P2 Receptor gene expression and prognostic difference was assessed by Kaplan-Meier plots and log-rank test [31]. Cox proportional hazard model analysis was done on the Korean patient cohort to test the interaction between HBV status and P2 purinergic receptor expression on the risk of recurrence.

## SUPPLEMENTARY MATERIAL FIGURES AND TABLES


