# Supplementary material for: P2X3 purinergic receptor overexpression is associated with poor recurrence-free survival in hepatocellular carcinoma patients
Source: Oncotarget. 2015 Oct 26;6(38):41162–79. doi: 10.18632/oncotarget.6240 (PMC4747397; doi:10.18632/oncotarget.6240)
Supplement: Supplementary file 1 [file oncotarget-06-41162-s001.pdf]

P2X3 purinergic receptor overexpression is associated with poor recurrence-free survival in hepatocellular carcinoma patients

Supplementary Material

Hepatocytes and HCC cell Lines. Normal human primary hepatocytes isolated from healthy adults (no known history of HCC), in suspension or freshly-frozen prior to shipment (Cryoport Systems, CA) were purchased from Triangle Research Labs, NC. Fresh hepatocytes were plated on collagen-coated tissue culture plates or glass coverslips in Williams E complete media with additives (10% fetal bovine serum, 2 mM glutamine, 2.5 g/ml insulin, 4 ng/ml glucagon, 2.5 g/ml transferrin, 2.5 ng/ml sodium selenite, 10,000 U/ml penicillin, 10,000 g/ml streptomycin, 50 g/ml gentamycin) for 3 h to ensure hepatocyte adherence to plates. Subsequently, hepatocytes were maintained in Williams E minimal media free from serum and growth factors for 24 h prior to treatment. Human hepatocellular carcinoma derived Huh7, and Hep3B, were cultured in Minimum Essential Medium Eagle (MEM); SNU-387 was cultured in Roswell Park Memorial Institute (RPMI) medium and PLC/PRF/5 cultured in Dulbecco's Modified Eagle's medium (DMEM). All maintenance culture media were supplemented with 10% fetal bovine serum (FBS), L-Glutamine (2 mM), penicillin (100 units/ml) and streptomycin (100 mg/ml) at 37°C and 5% CO_2. Cells were maintained in serum free media (containing 2 mM L-Glutamine, 100 units/ml penicillin and (100 mg/ml)) streptomycin for 24 h prior to treatment.

Cell transfection. Huh7 cells were maintained in MEM with 10% fetal bovine serum (FBS), 5% L-Glutamine and 5% Penicillin-streptomycin overnight. P2X3 or pCMV6 vector control plasmids (1μg) were transfected with Turbofectin 8.00 (Origene Technologies, Rockville, MD) in MEM with 5% L-Glutamine. Media was replaced after 24h, according to manufacturer's instructions**.**

Immunohistochemistry. Formalin-fixed and paraffin embedded liver sections from HCC patients were analyzed by immunohistochemistry with anti-P2X3 antibody (Abcam, Cambridge, MA). HCC cells were grown on glass coverslips; BrdU (10 μM, Roche, Indianapolis, IN) was added to culture media for 1 h prior to fixation (cold acetone: methanol, 1:1) and stained using anti-BrdU antibody (DAKO, Carpinteria, CA) and DAB Peroxidase Substrate Kit (Vector Labs, Burlingame, CA), according to manufacturer's instructions. Counterstaining was done with hematoxylin. BrdU positive cells were counted and expressed as a percentage of the total number of cells in ten randomly selected high-power fields (20X) per coverslips.

MTT assay. Cell viability was determined using the 3-(4, 5-dimethylthiazol-2-yl)-2,5-diphenyltetrazolium (MTT, Sigma) assays. Cells were plated in 96-well plates, maintained in serum-free media for 24h and treated with ATP (100 μM) for 18h. Cell were exposed to MTT solution (0.7 mg / ml) and incubated at 37 °C for 2 h. The media was removed and 200 μl of dimethyl sulphoxide (DMSO) was added to each well. After shaking the plates for 30 min, the absorbance at 570 nM was measured (background subtraction at 650 nM).

Western blotting. Total protein extracts were obtained by homogenizing cells in total lysis buffer (50 mM Tris-HCl, pH 7.5, 0.5 M NaCl, 2 mM EGTA, 2 mM EDTA, 1.0% Triton X-100, 0.25% Deoxycholate, 1 μg/ml pepstatin, 1.0 μg/ml leupeptin, 1.0 μg/ml aprotinin, 1.0 mM

phenylmethylsulfonyl fluoride, 1.0 mM Dithiothreitol, 2.0 mM activated Na_3VO_4, 2.0 mM NaF) and centrifuging at 14,000 rpm for 10 min (4°C). Equal amounts of total proteins as determined by BCA protein Assay (Pierce, Rockford, IL) were analyzed by Western blotting as described previously (9). Blots were probed with antibody specific for α-tubulin or GAPDH to ensure equal loading of proteins in each lane.

Real-time quantitative Reverse-Transcriptase Polymerase Chain Reaction (qRT-PCR). Total RNA was isolated from human livers or cells using Trizol Reagent according to manufacturer's instructions (Invitrogen, NY). Complementary DNA (cDNA) synthesis was performed by reverse transcription of total RNA (2 μg) with high capacity cDNA reverse transcription kit (Applied Biosystems, Foster city, CA). The cDNA product was amplified by qRT-PCR in Step One Plus Real-Time PCR system using SYBR Green PCR Master Mix (Applied Biosystems, Grand Island, NY). Quantitative expression values were determined using the $\Delta\Delta C_t$ method as specified by manufacturers using GAPDH as a control. DNA sequences of gene-specific primers are listed in Suppl. Table 3.

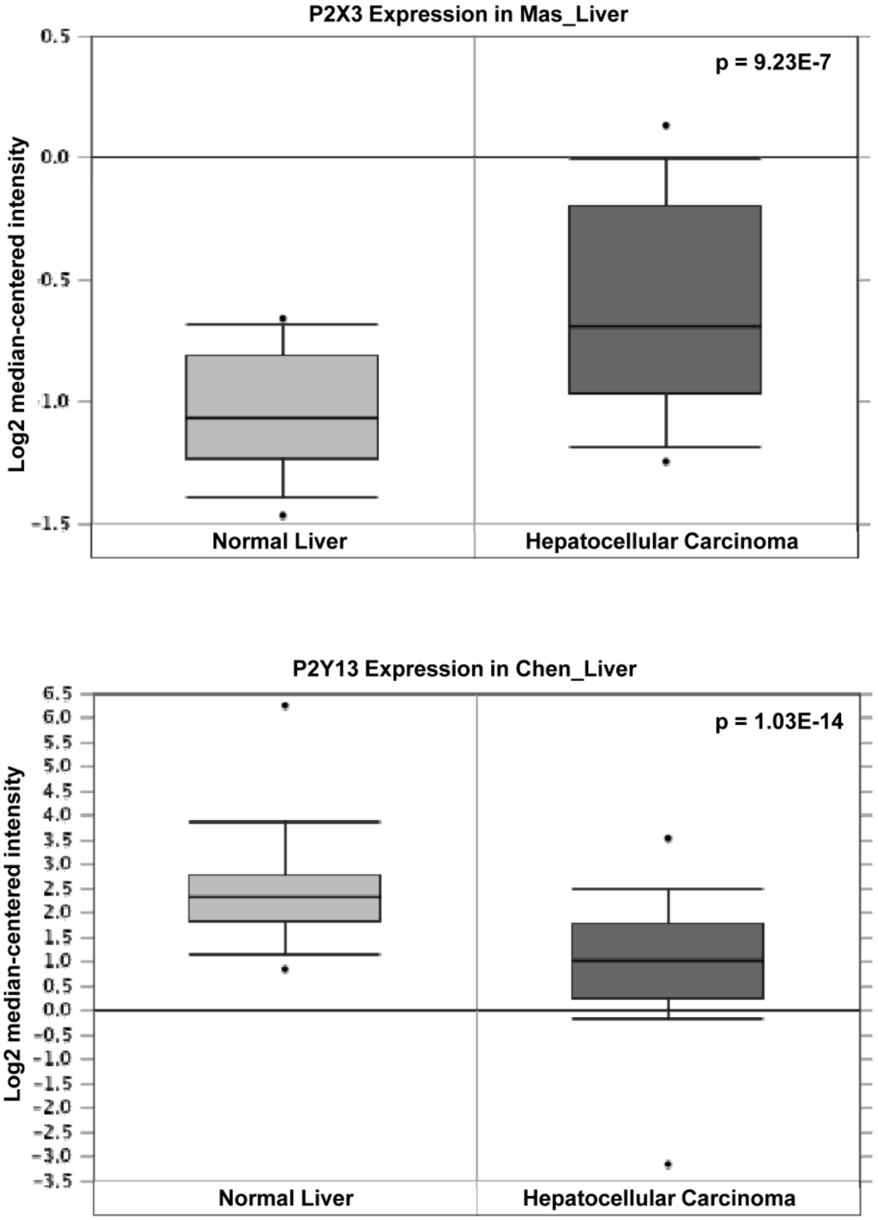

Suppl. Figure 1. Oncomine analysis of P2X3 (Mas Liver dataset) and P2Y13 (Chen Liver dataset) mRNA expression in Hepatocellular Carcinoma vs normal liver. Data represents log2 median-centered intensity ± SD.

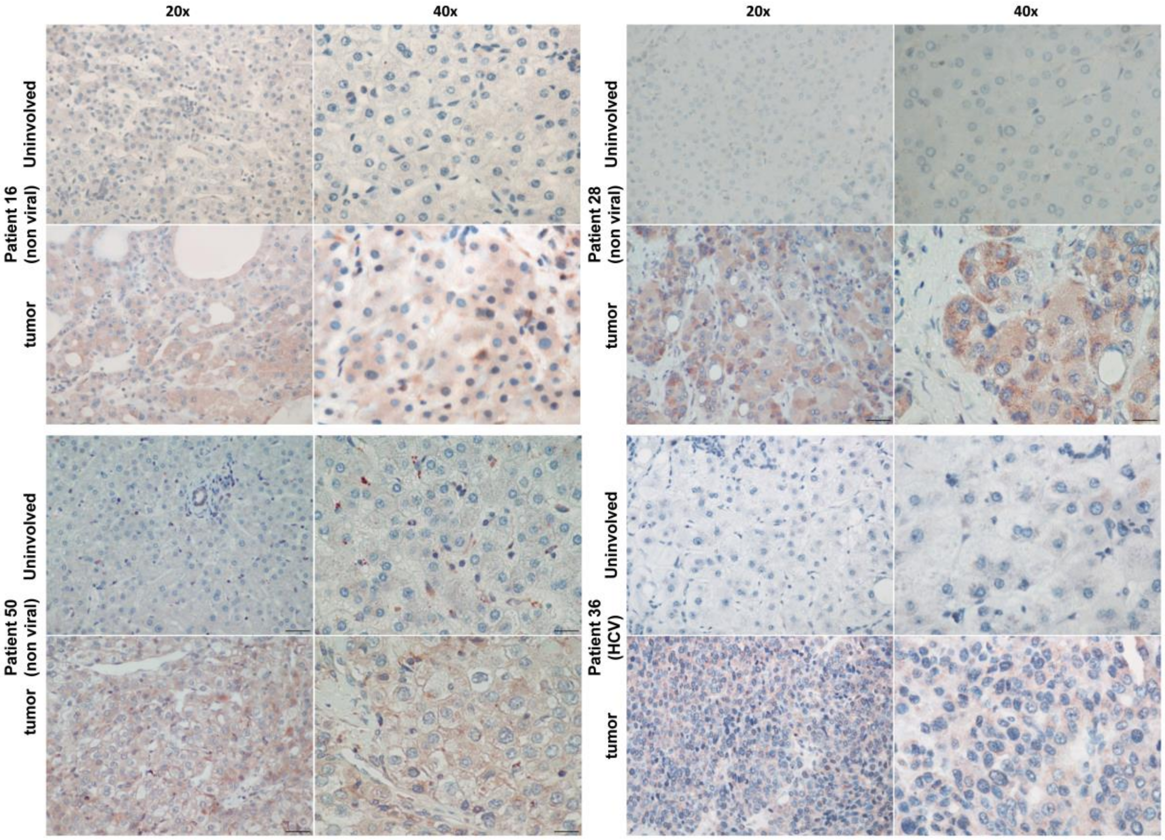

Suppl. Figure 2. Increased P2X3 purinergic receptor protein expression in HCC tumors with viral and non-viral etiologies. Immunohistochemical analysis of P2X3 expression in TMC cohort liver tumors.

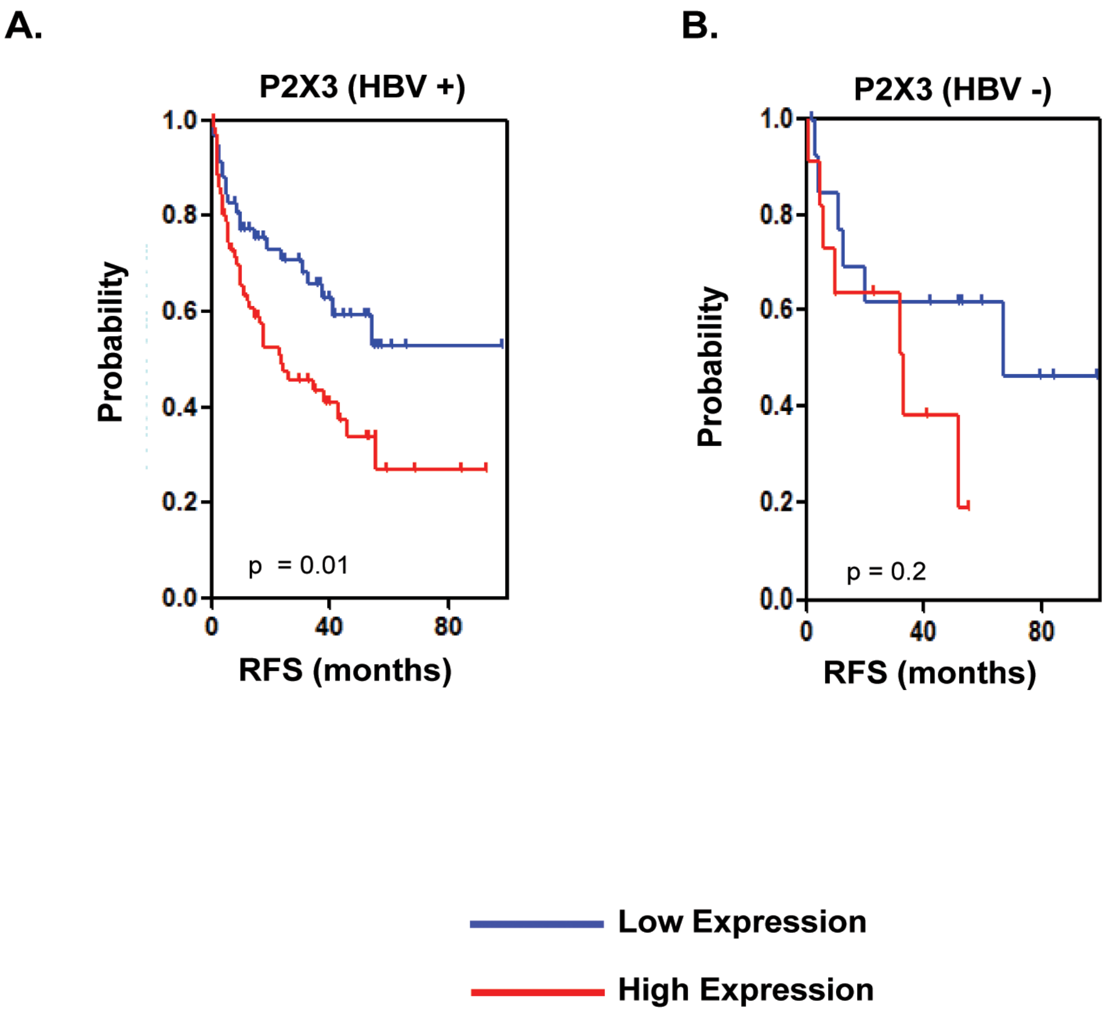

Suppl. Figure 3. Increased P2X3 purinergic receptor mRNA expression is associated with poor recurrence-free survival regardless of HBV status. Recurrence-free survival analysis (Kaplan-Meier) of Korean patient cohort; A) HBV positive - 'low' P2X3 (below median) vs 'high P2X3 (above median) n=131 and B) HBV negative - 'low' P2X3 (below median) vs 'high P2X3 (above median) n=25.

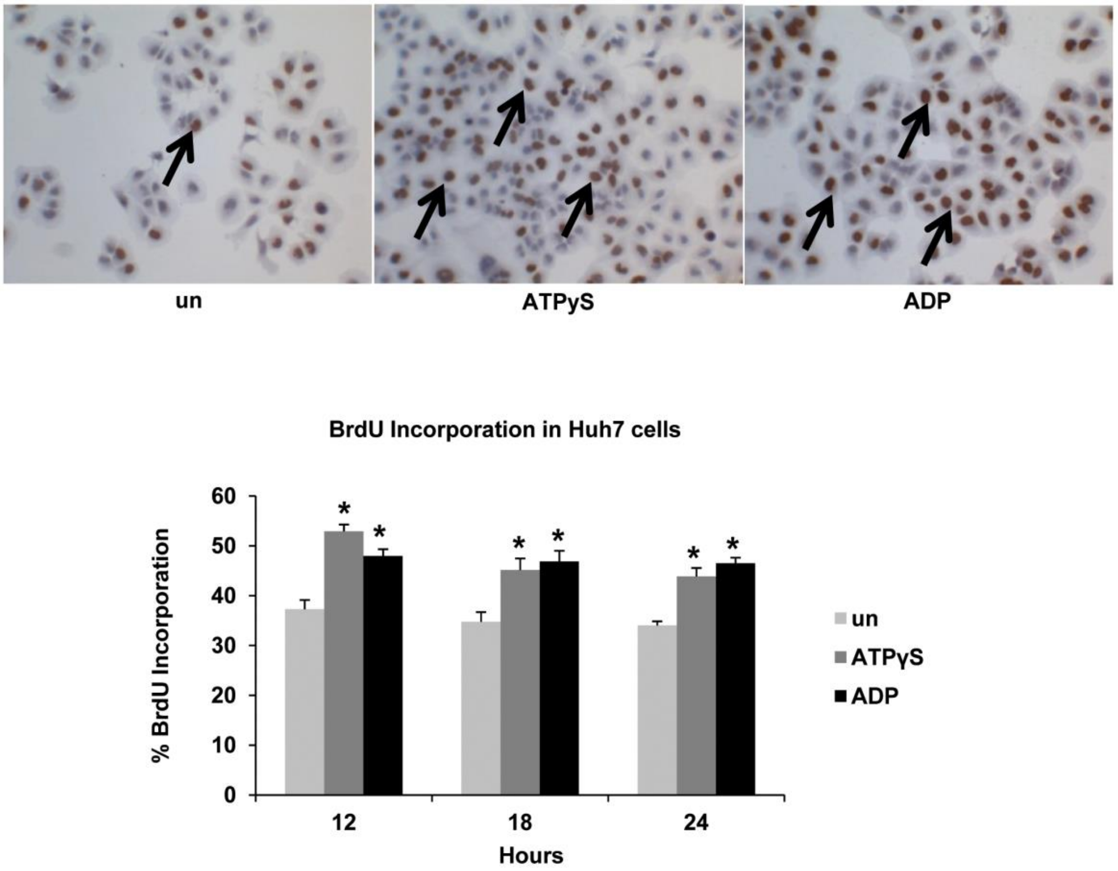

Suppl. Figure 4. Extracellular nucleotides induce proliferation in Huh7 cells *in vitro*. Huh 7 cells were maintained in serum-free conditions for 24 h prior to treatment with ATPγS or ADP for 12, 18 and 24 h. Light microscopic images (10X) of BrdU immunostained cells. BrdU-positive cells are expressed as a percentage of total number of cells. Data represents mean ± SEM, n=3-6, *$p<0.05$ vs untreated (un).

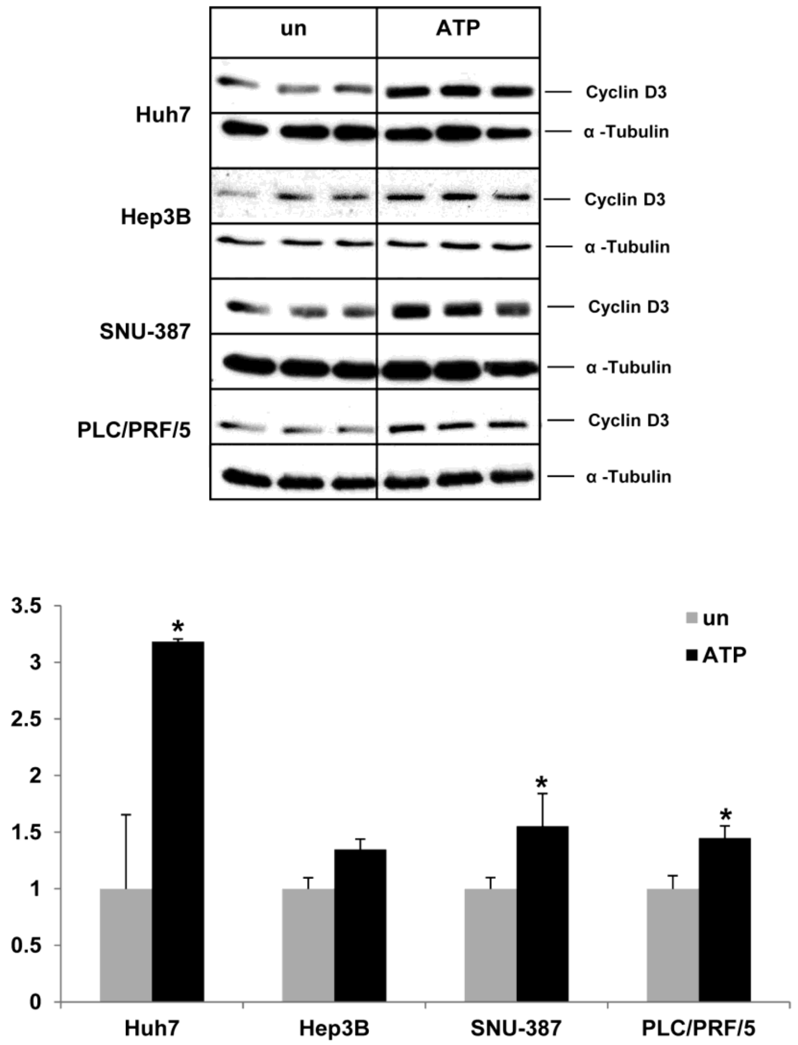

Suppl. Figure 5. Extracellular ATP treatment induces cyclin D3 protein expression in HCC cells *in vitro*. HCC cells (Huh7, Hep3B, SNU-387, and PLC/PRF/5) maintained in serum-free conditions were treated with ATP for 24 h and total protein extracts were analyzed by Western blotting for cyclin D3 expression. α-tubulin, protein loading control. Data represents mean ± SD, n=3-6, *p<0.05 vs untreated (un).

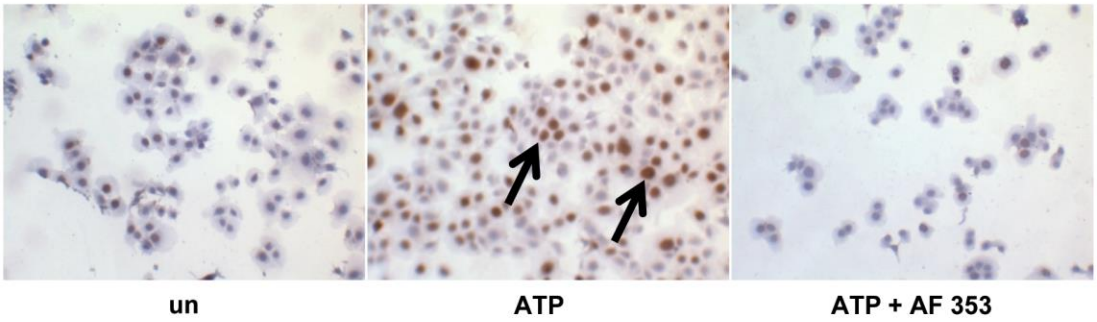

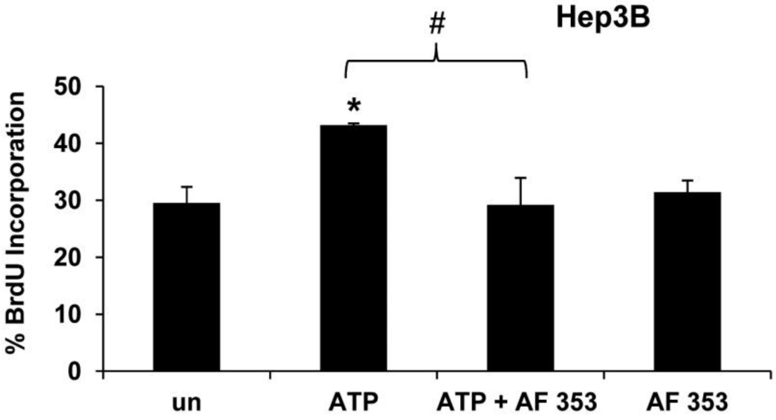

Suppl. Figure 6. P2X3 antagonist, AF-353, attenuates ATP-mediated activation of Hep 3B cell proliferation, *in vitro*. Light microscopic images (10X) of BrdU immunostained cells. Hep3B cells were maintained in serum-free conditions for 24 h and were pre-treated with AF-353 (5 μM) for 30 min, prior to treatment with ATP (100 μM) for 24 h. BrdU-positive cells are expressed as a percentage of total number of cells. Data represents mean ± SEM, n=3-6, *p<0.05 vs untreated (un).

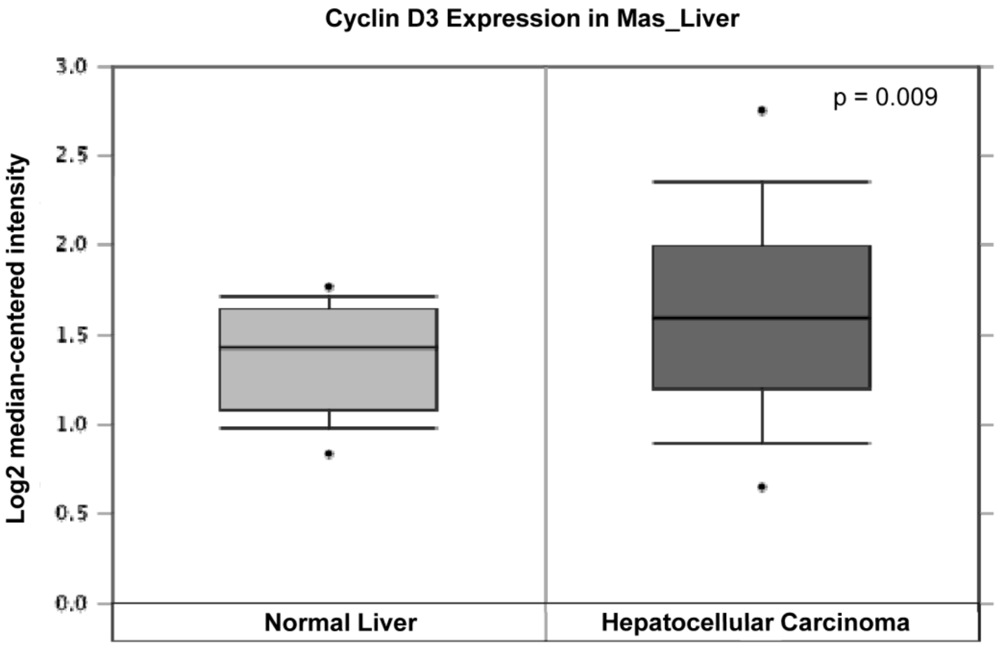

Suppl. Figure 7. Oncomine analysis of Cyclin D3 (Mas Liver dataset) mRNA expression in Hepatocellular Carcinoma vs normal liver. Data represents log2 median-centered intensity ± SD.

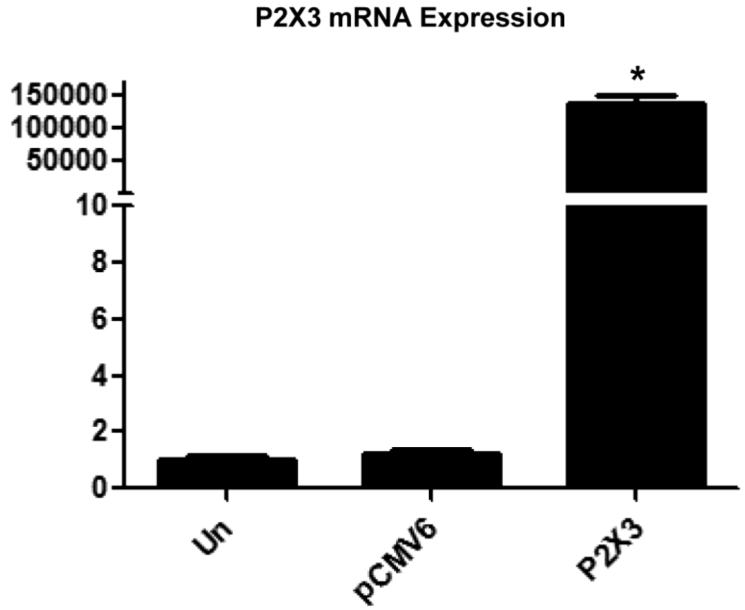

Suppl. Figure 8. Overexpression of P2X3 RNA in Huh7 cells. RNA isolated from Huh7 cell after transfection with P2X3 DNA or pCMV6 vector control plasmids (1µg) for 24h were analyzed by qRT-PCR for P2X3 mRNA. Data represented as the mean ± SEM, n=4, *$p < 0.05$ vs. untreated.

Supplementary Table 1. Clinical and pathological features of HCC patients (TMC cohort)

Variable		TMC Cohort
Number of Patients		42
	male	24 (57%)
	female	12 (29%)
	NA	6 (14%)
Age	Median	57 y
	Range	14-76 y
Viral status	HCV	21 (50%)
	HBV	5 (12%)
	non viral	10 (24%)
	NA	6 (14%)
Cirrhosis	Yes	25 (60%)
	No	9 (21%)
	NA	8 (19%)
Vasculature Invasion	Yes	8 (19%)
	No	24 (57%)
	NA	10 (24%)
Histological Grade	Well differentiated	3 (7%)
	Well to moderately differentiated	6 (14%)
	Moderately differentiated	18 (43%)
	Moderately to poorly differentiated	3 (7%)
	Poorly differentiated	1 (2%)
	Undifferentiated	0 (0%)
	NA	11 (26%)
AJCC Stage	I	12 (29%)
	II	15 (36%)
	III	5 (12%)
	IV	0 (0%)
	NA	10 (24%)

AJCC, American Joint Committee on Cancer*;* HBV, hepatitis B virus; HCV, hepatitis C virus; NA, data not available.

Supplementary Table 2. Clinical and pathological features of HCC patients (Korean Cohort)

Variable	Korean Cohort
Number of patients	188
Sex, no. (%)	
Male	156 (83%)
Female	32 (17%)
NA	
Age at baseline, median (range)	56 y (25-77 y)
AFP >300 ng/ml at baseline, no. (%)	
Yes	55 (29%)
No	132 (70%)
NA	1 (1%)
HBV at baseline, no. (%)	
Yes	131 (70%)
No	25 (13 %)
NA	32 (17%)
AJCC stage at baseline, no. (%)	
I	103 (55%)
II	30 (16%)
III	55 (29%)
IV	0 (0%)
NA	
BCLC stage at baseline, no. (%)	
0	4 (2%)
A	106 (56%)
B	63 (34%)
C	11 (6%)
D	4 (2%)
NA	
Number of deaths	60
Median follow-up time	39.6 mo

AJCC, American Joint Committee on Cancer; AFP, α-fetoprotein; BCLC, Barcelona Clinic Liver Cancer; HBV, hepatitis B virus; NA, data not available.

Supplementary Table 3.

Gene	Forward Sequence	Reverse Sequence
GAPDH	5'-CGGAGTCAACGGATTTGGTCGTAT-3'	3'-AGCCTTCTCCATGGTGGTGAAGAC-3'
P2X1	5'-CGCCTTCCTCTTCGAGTATGA-3'	5'-AGATAACGCCCACCTTCTTATTACG-3'
P2X2	5'-GCCTACGGGATCCGCATT-3'	5'-TGGTGGGAATCAGGCTGAAC-3'
P2X3	5'-GCTGGACCATCGGGATCA-3'	5'-GAAAACCCACCCTACAAAGTAGGA-3'
P2X4	5'-CCTCTGCTTGCCCAGGTACTC-3'	5'-CCAGGAGATACGTTGTGCTCAA-3'
P2X5	5'-CTGCCTGTCGCTGTTCGA-3'	5'-GCAGGCCCACCTTCTTGTT-3'
P2X6	5'-AGGCCAGTGTGTGGTGTTCA-3'	5'-TCTCCACTGGGCACCAACTC-3'
P2X7	5'-TCTTCGTGATGACAAACTTTCTCAA-3'	5'-GTCCTGCGGGTGGGATACT-3'
P2Y1	5'-CGTGCTGGTGTGGCTCATT-3'	5'-GGACCCCGGTACCTGAGTAGA-3'
P2Y2	5'-GAACTGACATGCAGAGGATAGAAGAT-3'	5'-GCCGGCGTGGACTCTGT-3'
P2Y4	5'-CCGTCCTGTGCCATGACA-3'	5'-TGACCGCCGAGCTGAAGT-3'
P2Y6	5'-GCCGGCGACCACATGA-3'	5'-GACCCTGCCTCTGCCATTT-3'
P2Y11	5'-CTGGAGCGCTTCCTCTTCAC-3'	5'-GGTAGCGGTTGAGGCTGATG-3'
P2Y12	5'-AGGTCCTCTTCCCACTGCTCTA-3'	5'-CATCGCCAGGCCATTTGT-3'
P2Y13	5'-GAGACACTCGGATAGTACAGCTGGTA-3'	5'-GCAGGATGCCGGTCAAGA-3'
P2Y14	5'-TTCCTTTCAAGATCCTTGGTGACT-3'	5'-GCAGAGACCCTGCACACAAA-3'
Cyclin D3	5'-AGGGATCACTGGCACTGAAG-3'	5'-ACAGGTGTATGGCTGTGACAT-3'
Cyclin E	5'-TGTGTCCTGGATGTTGACTGCC-3'	5'-CTCTATGTCGCACCACTGATACC-3'
Cyclin A	5'-GCACACTCAAGTCAGACCTGCA-3'	5'-ATCACATCTGTGCCAAGACTGGA-3'
Cyclin B	5'-GACCTGTGTCAGGCTTTCTCTG-3'	5'-GGTATTTTGGTCTGACTGCTTGC-3'
